# A case of hepato-biliary infection secondary to cryptosporidium in a patient on tacrolimus

**DOI:** 10.1099/jmmcr.0.005159

**Published:** 2018-07-20

**Authors:** Sajal Gupta, Alison Johnson, Simon Meyrick, Angharad P. Davies, R. Chalmers

**Affiliations:** ^1^​ Wye Valley NHS Trust, The County Hospital, Union Walk, Hereford, Herefordshire, HR1 2ER, UK; ^2^​ Cryptosporidium Reference Unit, Public Health Wales Microbiology ABM, Singleton Hospital, Sgeti, Swansea, SA2 8QA, UK

**Keywords:** cryptosporidium, tacrolimus, hepato-biliary, nitazoxanide

## Abstract

**Introduction.:**

Cryptosporidium infection is known to cause hepato-biliary involvement, mainly in association with T-cell immune deficiency. Hepato-biliary involvement in association with milder immunosuppression is less well described. We describe the first case, to our knowledge, of *Cryptosporidium hominis* hepato-biliary infection associated with tacrolimus in a patient with nephrotic syndrome.

**Case presentation.:**

A 14 year old girl who had been on tacrolimus for nephrotic syndrome presented with diarrhea due to *C. hominis*. Nineteen days after her initial presentation she attended hospital with abdominal pain and deranged liver function tests. An ultrasound scan showed a thickened gall bladder. Her symptoms settled and her liver function tests returned to normal after treatment with nitazoxanide.

**Conclusion.:**

Cryptosporidium should be considered in the differential diagnosis of both diarrhea and hepato-biliary symptoms and abnormal liver function tests, even in the presence of relatively mild immunosuppression. Nitazoxanide was an effective treatment in this case.

## Introduction

Cryptosporidium is a protozoan parasite and is a common cause of diarrhoea. It can cause chronic diarrhoea in immunocompromised patients and this can be associated with pancreato-biliary infection. This severe manifestation of the infection is most frequently associated with severe T cell deficiency, such as is seen in HIV infection, with CD4 counts lower than 200; in haematological malignancies and in patients with primary T cell deficiencies [1].

We present a case of biliary involvement secondary to cryptosporidium infection in a patient on relatively mild immunosuppressive treatment whose symptoms resolved following treatment with nitazoxanide.

## Case report

A 14 year old girl developed diarrhoea and vomiting and presented to hospital four days after symptom onset following a fainting episode. She had also experienced sharp colicky pains in her abdomen. She had been on holiday in the United Kingdom prior to the episode.

The patient had developed nephrotic syndrome at age 13 with a renal biopsy showing focal segmental glomerulosclerosis. She also had mild asthma, menorrhagia and pulmonary stenosis (diagnosed on echocardiogram).

Her medications included tacrolimus, enalapril, atrovastatin, omeprazole, penicillin-V, levothyroxine and ferrous fumarate. The tacrolimus had been stopped a day before the start of illness as it had not produced any improvement in renal function or reduction in proteinuria. The tacrolimus had been commenced 14 months earlier at a dose of 5 mg twice daily. A tacrolimus level had last been assayed 2 months before onset of her illness. This was 7.3 µg l^−1^ i.e. within the normal therapeutic range.

On admission, she was felt to be fluid-depleted with mild dehydration and after initial treatment with a bolus of 500 ml of normal saline in the emergency department, was commenced on intravenous fluids in view of abdominal pain and vomiting. Her renal function was deranged with an acute kidney injury score of 2. Urea was 22.8 mmol l^−1^, previously having being in the range of 6.5–11.8 mmol l^−1^ and creatinine was raised to 157 µmol l^−1^, previously having being in the range of 70–95 µmol l^−1^. Liver function tests were normal. White blood cell count was normal (6.1×10^9^ l^−1^). Enalapril was stopped in view of these results. She improved after 24 h of maintenance IV fluid therapy with 0.9 % saline and 5 % dextrose, renal function returning to previous levels (urea 16.7 mmol l^−1^, Creatinine 94 µmol l^−1^) and she was discharged. Subsequently, *Cryptosporidium* was detected on stool microscopy. This was confirmed to be *Cryptosporidium hominis* by PCR at the national reference laboratory. The sample was negative for all other pathogens tested for, including *Escherichia coli* 0157, *Campylobacter*, *Salmonella* and *Shigella*. Following discharge she continued to have some episodes of diarrhoea but no vomiting. She re-presented 19 days later with significant abdominal pain and infrequent small vomits which were non bilious, her diarrhoea had resolved.

On examination she had epigastric tenderness but no hepatomegaly. Her perfusion was adequate with a capillary refill time <2 s, pulse rate of 89, blood pressure was 104/58, both within the normal range for her age. However, her peripheral pulses were poor. She was commenced on maintenance intravenous fluids with 0.9 % saline and 5 % dextrose. Liver function tests were noted to be deranged with raised aspartate transaminase (451 IU l^−1^), alanine transaminase (267 IU l^−1^), gamma-glutamyl transferase (115 IU l^−1^) and lactate dehydrogenase (853 IU l^−1^). Her renal function showed raised urea (10 mmol l^−1^) and creatinine (72 µmol l^−1^) which were above baseline but within her usual range. Her liver function continued to deteriorate over the next 24 h (peak aspartate transaminase 1062 IU l^−1^, alanine transaminase 1292 IU l^−1^, lactate dehydrogenase 882 IU l^−1^, gamma-glutamyl transferase 209 IU l^−1^). An ultrasound showed a thickened gall bladder but otherwise normal liver and bile duct.

After discussing the history and results with specialist paediatric hepatologists who, in view of the facts, that her stool was positive for *Cryptosporidium hominis,* she had worsening liver function tests and ultrasound evidence of cholangitis, recommended a three day oral course of nitazoxanide 500 mg twice daily. Following this, her liver function tests rapidly improved, returning almost to baseline after 4 days. Her appetite returned to normal and her abdominal symptoms resolved.

## Discussion

*Cryptosporidium* infection should be considered in the differential diagnosis of both diarrhoea and hepato-biliary symptoms and abnormal liver function tests even in the presence of relatively mild immunosuppression.

The biliary system provides a reservoir for the *Cryptosporidium* parasite and cryptosporidiosis of the pancreato-biliary system is well-recognised in patients who are immune-compromised, especially in those with T-cell immune deficiency [2]. Sclerosing cholangitis as a result is well-described [3–6]. *Cryptosporidium* enteritis in solid organ transplant recipients has previously been associated with elevated tacrolimus concentrations [7] and there also exists one report of *C. parvum*-induced sclerosing cholangitis in an adult renal transplant patient taking tacrolimus [8]. To the best of our knowledge, this is the first report of *C. hominis* associated with use of tacrolimus in a patient with nephrotic syndrome. Clinicians should bear in mind the risk of cryptosporidiosis, including of the biliary tract, in patients taking tacrolimus.

Nitazoxanide is not licensed for treatment of cryptosporidiosis in the UK but is available on a named patient basis. It produces an improvement in diarrhoea in immunocompetent children [9] and in HIV patients with CD4 counts over 50 [10]. It produced an excellent symptom response in our patient with presumed hepato-biliary cryptosporidiosis and relatively minor immunosuppression. The suspension of tacrolimus probably also contributed to the resolution of symptoms by allowing resumption of the normal immune response.
